# Revealing the Selective Bifunctional Electrocatalytic Sites via In Situ Irradiated X‐Ray Photoelectron Spectroscopy for Lithium–Sulfur Battery

**DOI:** 10.1002/advs.202206786

**Published:** 2023-01-16

**Authors:** Pengpeng Zhang, Yige Zhao, Yukun Li, Neng Li, S. Ravi P. Silva, Guosheng Shao, Peng Zhang

**Affiliations:** ^1^ State Centre for International Cooperation on Designer Low‐Carbon and Environmental Materials (CDLCEM) Zhengzhou University 100 Kexue Avenue Zhengzhou 450001 China; ^2^ Zhengzhou Materials Genome Institute (ZMGI) Zhengzhou Zhengzhou 450001 China; ^3^ State Key Laboratory of Silicate Materials for Architecture Wuhan University of Technology Wuhan 430000 China; ^4^ Nanoelectronics Center Advanced Technology Institute University of Surrey Guildford GU27XH UK

**Keywords:** directly observation, electrocatalytic sites, in situ irradiation X‐ray photoelectron spectroscopy, light field, selective bifunctional electrocatalyst

## Abstract

The electrocatalysts are widely applied in lithium–sulfur (Li–S) batteries to selectively accelerate the redox kinetics behavior of Li_2_S, in which bifunctional active sites are established, thereby improving the electrochemical performance of the battery. Considering that the Li–S battery is a complex closed “black box” system, the internal redox reaction routes and active sites cannot be directly observed and monitored especially due to the distribution of potential active‐site structures and their dynamic reconstruction. Empirical evidence demonstrates that traditional electrochemical test methods and theoretical calculations only probe the net result of multi‐factors on an average and whole scale. Herein, based on the amorphous TiO_2‐_ *_x_* @Ni selective bifunctional model catalyst, these limitations are overcome by developing a system that couples the light field and in situ irradiated X‐ray photoelectron spectroscopy to synergistically convert the “black box” battery into a “see‐through” battery for direct observation of the charge transportation, thus revealing that amorphous TiO_2‐_ *_x_* and Ni nanoparticle as the oxidation and reduction sites selectively promote the decomposition and nucleation of Li_2_S, respectively. This work provides a universal method to achieve a deeper mechanistic understanding of bidirectional sulfur electrochemistry.

## Graphical abstract

In situ irradiated X‐ray photoelectron spectroscopy coupled with the light field is hired to synergistically convert the “black box” battery into a “see‐through” battery for direct observation of the charge transportation, thus revealing that amorphous TiO_2‐_ *_x_* and Ni nanoparticle as the oxidation and reduction sites selectively promote the decomposition and nucleation of Li_2_S, respectively.

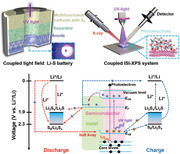

## 1 Introduction

Lithium–sulfur (Li–S) batteries are attracting much attention as the puissant candidates of next‐generation energy storage devices due to their high theoretical energy density (2600 Wh kg^−1^) and environmental friendliness.^[^ ^1^, ^2^, ^3^, ^4^, ^5^, ^6^ ^]^ Nonetheless, Li–S batteries are still a long way from commercialization until now unless the dilemmas, including sluggish redox kinetics, severe “shuttle effect,” and large volume expansion, are resolved.^[^ ^7^, ^8^, ^9^ ^]^ Expediting the electrocatalytic transformation and mitigating the lithium polysulfides (LiPSs) accumulation are of vital importance in the Li–S system.^[^ ^10^, ^11^, ^12^ ^]^ Specifically, the redox process of S species involves the following multiple reactions in bidirectional sulfur electrochemistry

(1) $$\begin{array}{ccc} & & \;S_{8}\overset{+2e^{-}}{\to }{\mathrm{Li}}_{2}S_{8}^{*}\overset{+\frac{2}{3}e^{-}}{\to }\frac{4}{3}{\mathrm{Li}}_{2}S_{6}^{*}\overset{+\frac{4}{3}e^{-}}{\to }2{\mathrm{Li}}_{2}S_{4}^{*}\\ & & \;\;\overset{+4e^{-}}{\to }4{\mathrm{Li}}_{2}S_{2}\overset{+8e^{-}}{\to }8{\mathrm{Li}}_{2}S \end{array}$$

(2) $$\begin{array}{ccc} & & \;8{\mathrm{Li}}_{2}S\overset{-me^{-}}{\to }{\mathrm{Li}}_{2}S_{x}^{*}\left(x\;=\;4,\;6,\;8\right)\overset{-ne^{-}}{\to }S_{8}\\ & & \;\;\left(m\;+\;n=16\right) \end{array}$$

Although most of the theoretical capacity comes from the contribution of the conversion from LiPSs to Li_2_S, this process is slow and regarded as the rate‐determining step in the sulfur reduction reaction (SRR).^[^ ^13^, ^14^, ^15^, ^16^ ^]^ Even worse, the insulating Li_2_S tends to aggregate on the surface of the electrocatalyst, thus suppressing the catalytic activity. In addition, during the sulfur evolution reaction (SER), the high dissociation energy barrier of Li_2_S hampers its decomposition. Therefore, optimal Li_2_S manipulation demands both robust reduction of LiPSs and efficient dissociation of Li_2_S.^[^ ^17^, ^18^, ^19^, ^20^, ^21^ ^]^ Generally, the single functional electrocatalyst cannot satisfy the demands of complex multi‐step redox reactions in bidirectional.^[^ ^7^, ^14^, ^22^ ^]^ It is a matter, of course, to construct multifunctional electrocatalysts with a well‐defined division of labor and selective electrocatalytic pair sites. Therefore, some works adopted the strategy of multifunctional electrocatalysts to selectively accelerate the reduction reaction of LiPSs and the oxidation of Li_2_S, synergistically improve the electrochemical performance of lithium–sulfur batteries.^[^ ^23^, ^24^, ^25^, ^26^ ^]^ Furthermore, other strategies precisely regulated the critical and rate‐determining steps of the complex multiple redox reactions inside Li–S batteries to optimize the electrocatalytic kinetics.^[^ ^27^, ^28^, ^29^ ^]^ For example, Yang et al. used Indium (In)‐based electrocatalyst to selectively decelerate the solid–liquid conversion and accelerate the liquid–solid conversion in the SRR, thus inhibiting the shuttle effect.^[^ ^29^ ^]^

Despite the remarkable progress in regulating the redox kinetics of Li_2_S by manipulating the suitable configuration of electrocatalyst, since the Li–S battery is a complex closed “black box” system, the internal redox reaction routes and electrocatalytic sites cannot be directly observed and monitored especially due to the distribution of potential active‐site structures and their dynamic reconstruction.^[^ ^30^ ^]^ In the “black box” battery, empirical evidence demonstrates that traditional electrochemical test methods and theoretical calculations only probe the net result of multi‐factors in average and whole scale.^[^ ^31^, ^32^, ^33^, ^34^, ^35^, ^36^ ^]^ First, if we could open a window on top of this “black box” battery and introduce a physical field, such as the light field, the photogenerated electron‐hole pairs generated by the semiconductor electrocatalysts under solar light illumination would enhance the redox kinetics of Li_2_S and amplify the effect of the electrocatalytic process. Second, if we could couple an in situ characterization system to directly detect the charge transfer during the catalytic reaction, the distribution of potential electrocatalytic sites would be further confirmed. In situ irradiated X‐ray photoelectron spectroscopy (ISI‐XPS) was first employed for the investigation of the charge transfer pathway of photogenerated electrons by our group,^[^ ^37^, ^38^, ^39^, ^40^ ^]^ which will satisfy this requirement because it can illustrate whether a particular component in the catalysts gains or loses electrons by comparing the binding energies of corresponding elements with and without light excitation during the testing process.^[^ ^37^ ^]^ Therefore, it is challenging but desirable to develop a system that couples the additional physics gain effect with in situ characterization technique to synergistically convert the “black box” battery into a “see‐through” battery for direct observation of the electrocatalytic reaction routes and sites.

In this work, the amorphous TiO_2‐_ *_x_* @Ni‐CNF as a model catalyst was designed with selective electrocatalytic sites. Theoretical calculations and electrochemical evidence demonstrate that the Ni nanoparticle can not only effectively trap dissolved LiPSs but also catalyze the nucleation of Li_2_S during the SRR, while the amorphous TiO_2‐_ *_x_* is mainly responsible for accelerating the decomposition of insoluble Li_2_S during the charging process. More importantly, under light irradiation, the photogenerated carriers generated by the electrocatalyst are injected into the Li–S battery and amplify the electrocatalytic reaction. Subsequently, the introduction of ISI‐XPS allows us to directly observe the charge transport in the amorphous TiO_2‐_ *_x_* @Ni heterojunction, thus revealing that amorphous TiO_2‐_ *_x_* and Ni nanoparticle as the oxidation and reduction sites selectively promote the decomposition and nucleation of Li_2_S, respectively. The Li–S battery using this electrocatalyst delivers a remarkable initial area capacity of 12.1 mAh cm^−2^ with a high sulfur loading of 11.7 mg cm^−2^ and lean electrolyte/sulfur ratio of 5 µL mg^−1^. Impressively, the pouch cell with S/NTCNF cathode exhibits outstanding flexibility and can still operate continuously under harsh working conditions. This work demonstrates a feasible strategy for the construction of bifunctional electrocatalysts with selective pair sites that can selectively catalyze the reduction of LiPSs and the decomposition of Li_2_S and provides a universal method to achieve a deeper mechanistic understanding of the bidirectional sulfur electrochemistry.

## 2 Results and Discussion

**Scheme 1:**
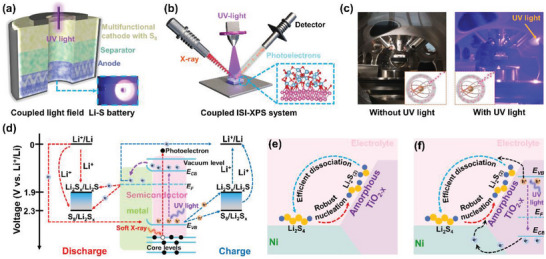
a) Configuration of the coupled light field Li–S battery (blue dashed box: digital photograph). b) Schematic illustrations of the working configuration of ISI‐XPS. c) Digital photograph of ISI‐XPS device. Inset: schematic diagram of the shielding effect. d) Proposed diagram of charge transfers of photogenerated carriers in the Li–S battery and working mechanism of ISI‐XPS. e) The optimal redox mechanism with amorphous TiO_2‐_ *_x_* @Ni selective bifunctional electrocatalyst in the traditional Li–S configuration. f) The enhanced kinetics of the redox reaction with amorphous TiO_2‐_ *_x_* @Ni selective bifunctional electrocatalyst in the coupled light field Li–S battery.

**Scheme** **1**a–c presents the schematic illustration and digital photographs of the coupled light field Li–S battery and ISI‐XPS. Different from traditional Li–S batteries, the coupled light field Li–S battery has a transparent window sealed with PET tape, which transmits light and stabilizes the organic electrolyte (Scheme 1a). As supposed, ISI‐XPS will directly reveal the carrier migration paths in the electrocatalysts by detecting the binding energy (BE) variations of corresponding elements with and without UV light excitation (Scheme 1b). The digital photograph of the ISI‐XPS device is shown in Scheme 1c. As is well‐known, the photoelectric effect is the basis of the ISI‐XPS technique (Inset of Scheme 1c,d). Since the photoelectron receives not only the attraction from the nucleus but also the repulsion from outer electrons, the attraction of the nucleus to the photoelectron will be weakened as the density of outer electrons increases, thus causing increased kinetic energy (KE) of the photoelectrons excited by X‐ray. Hence, the increase of outer electrons will lead to the decrease of BE according to the photoelectric effect equation:

(3) KE=h−BE,

showing a blue shift in the XPS spectrum. Similarly, the decrease of outer electrons will result in a red shift of BE. To specify how photogenerated carriers participate in the redox reaction of sulfur species, the proposed diagram of charge transfers of photo‐generated electrons and holes in the Li–S battery under the solar light illumination is given in Scheme 1d. Since the Gibbs free energy of forming the constantly changing polysulfides by the sulfur reduction in the presence of lithium ions are very close, the reaction potentials of S_8_ and Li_2_S are chosen to fit the actual voltage range of 1.9–2.3 V. Apparently, the energy level of conduction band (CB) in the semiconductor is higher than the reduction potentials of S_8_/Li_2_S*_x_* and Li_2_S*_x_*/Li_2_S so that the photo‐generated electrons excited in the CB have enough reduction ability to gradually reduce S_8_ to long‐chain LiPSs, then shorten the sulfur chain and finally convert to Li_2_S in the discharge process. Simultaneously, the holes left in the valance band (VB) are reduced by the electrons from the external circuit.^[^ ^41^ ^]^ The energy level of VB is lower than the oxidation potentials of Li_2_S/Li_2_S*_x_* and Li_2_S*_x_*/S_8_, thus in the charge process, Li_2_S is gradually oxidized to Li_2_S*_x_* driven by the photo‐generated holes in the VB, accompanied by pushing the photo‐generated electrons to the anode via the external circuit with the applied charging voltage, and reducing Li‐ion to Li. Therefore, the redox kinetics of sulfur can be highly promoted during the whole process, and the solar energy can be injected into the Li–S battery as well, realizing the light gain effect to amplify the electrocatalytic reactions. As shown in Scheme 1e, the designed Ni nanoparticle and amorphous TiO_2‐_ *_x_* pair sites selectively catalyze the reduction of Li_2_S_4_ and accelerate the oxidation of Li_2_S in the general Li–S system. With the injection of solar light energy, the photo‐generated electrons in the CB of amorphous TiO_2‐_ *_x_* will migrate to the surface of Ni nanoparticles to enhance the reduction reaction kinetics of LiPSs. The photogenerated holes left in the VB of amorphous TiO_2‐_ *_x_* will assist to accelerate the oxidation of Li_2_S during the charging process (Scheme 1f). If the directional transportation of photogenerated carriers in the amorphous TiO_2‐_ *_x_* @Ni heterojunction can be directly observed and confirmed, it can help to reveal the selective electrocatalytic pair sites and redox reaction route in the designed Li–S system.

**Figure 1:**
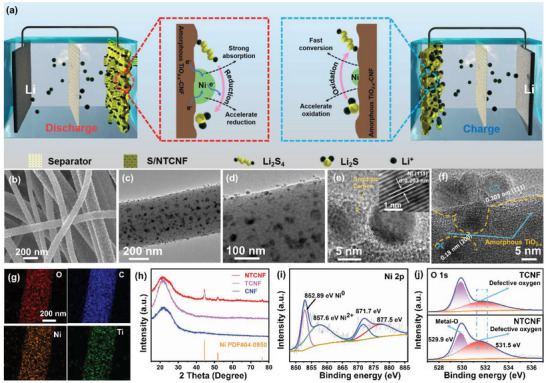
a) Selectively accelerating the Li_2_S_4_ reduction and Li_2_S oxidation mechanism with amorphous TiO_2‐_ *_x_* @Ni bifunctional electrocatalyst in the discharge and charge process of Li–S configuration. b) SEM image of NTCNF. c,d) TEM images of NTCNF. e,f) HRTEM images of NTCNF. Inset: the inverse FFT image corresponding to the amplified part of (e). g) The corresponding elemental mapping of O, C, Ni, and Ti of NTCNF. h) XRD patterns of NTCNF, TCNF, and CNF. i) XPS Ni 2p spectrum of NTCNF. j) XPS O 1s spectrum of NTCNF and TCNF.

Electrocatalyst design in the Li–S system frequently employs single functional electrocatalysts. However, it is insufficient for expediting the sulfur bidirectional reaction kinetics because the single functional electrocatalysts cannot balance the two opposing reaction types of reduction and oxidation. In comparison, the design and engineering of electrocatalysts with bifunctional pair sites allow us to selectively manipulate and optimize the bidirectional Li_2_S redox kinetics. Therefore, the proposed optimal Li_2_S manipulation system is shown in **Figure** **1**a. The Ni nanoparticles and amorphous TiO_2‐_ *_x_* pair sites anchored on the flexible electrospinning carbon nanofibers (CNFs) substrate promote Li_2_S nucleation and dissociation processes, respectively. The process of one‐step synthesis of flexible amorphous TiO_2‐_ *_x_* @Ni‐CNF (denoted as NTCNF) electrode materials by the electrospinning method is shown in Figure S1, Supporting Information. The precursors are prepared by dissolving tetrabutyl titanate (C_16_H_36_O_4_Ti), (CH_3_COOH)_2_·Ni·4H_2_O (nickel acetate tetrahydrate), and polyvinyl pyrrolidone (PVP) in ethanol (C_2_H_5_OH) solution for electrospinning. Acetic acid (CH_3_COOH) was added as a suppressant to inhibit the hydrolysis of tetrabutyl titanate. The as‐prepared CNFs were first stabilized in the air at 250 °C for 2 h and subsequently carbonized at 600 °C for 1 h in the Ar atmosphere to obtain the NTCNF host. As a contrast, the nickel acetate tetrahydrate salts were removed to prepare the amorphous TiO_2‐_ *_x_*‐CNF (denoted as TCNF) electrode. Pure CNFs were obtained by removing both nickel acetate tetrahydrate salts and tetrabutyl titanate. More synthesis details are described in the Experimental Section. The morphology of the as‐prepared samples was verified by scanning electron microscopy (SEM) and transmission electron microscopy (TEM). As shown in Figure S2a–d, Supporting Information, pure CNF is larger than one micron in diameter, after the introduction of the titanium ions, the diameter of TCNF materials was reduced to about 500 nm. It is worth noting that the surface of TCNF was coated with bark‐like amorphous components, which are considered to be amorphous TiO_2‐_ *_x_* and will be discussed below (Figure S2d, Supporting Information). From the SEM and TEM images (Figure 1b–d and Figure S3a, Supporting Information), the nickel nanoparticles are uniformly dispersed on the surface of NTCNF hosts with a diameter of about 10 nm, which is in strong contrast with TCNF. These 3D interconnected rough CNFs accompanied by a uniform distribution of nickel particles are favorable for the formation of Ni—S bonds during the reduction process of LiPSs to accelerate the nucleation of Li_2_S/Li_2_S_2_. The single‐crystalline Ni nanocrystalline corresponding to the (111) and (200) lattice plane was surrounded by several graphitic carbon layers,^[^ ^42^, ^43^ ^]^ which is confirmed by the HRTEM image in Figure 1e and Figure S3c,d, Supporting Information. Similarly, in Figure 1f, in addition to the lattice fringes corresponding to the (111) and (200) lattice plane of Ni nanoparticle, there is also a flocculent dark area connected with it, which is amorphous TiO_2‐_ *_x_*. These morphological results reveal that Ni nanoparticles are uniformly dispersed on the flexible 3D CNF framework with amorphous TiO_2‐_ *_x_* being coated on the fiber surface to form strong heterogeneous pair sites. Element mappings of the NTCNF electrode (Figure 1g and Figure S3b, Supporting Information) show the intact connection and uniform distribution of amorphous TiO_2‐_ *_x_* and Ni nanoparticle components. Crystallographic structure and chemical compositions of as‐prepared materials were explored by X‐ray diffraction (XRD) patterns. As shown in Figure 1h, there is no characteristic peak of TiO_2_ in the XRD patterns of TCNF. After the nickel element was loaded, only three distinct diffraction peaks appeared in NTCNF, which can be assigned to the (111), (200), and (220) lattice planes of the Ni element (PDF#04‐0850), respectively. In contrast, Ti and O elements can be clearly detected in the element mappings of NTCNF (Figure 1g). To further confirm the composition of titanium oxide, the synthesis temperature of NTCNF was raised to 700 °C and labeled as NTCNF‐700. As can be seen from Figure S4, Supporting Information, in the XRD pattern of NTCNF‐700, not only the characteristic peak intensity of Ni becomes higher, which usually represents the improvement of crystallinity, but also clear diffraction peaks of TiO_2_ (PDF#21‐1276) can be identified.

The surface chemistry phase and composition of the materials are examined via X‐ray photoelectron spectroscopy (XPS) measurements. The wide XPS spectra of NTCNF and TCNF materials are shown in Figures S5a and S6a, Supporting Information. As illustrated in Figure S5b, Supporting Information, there are two peaks at 458.3 and 463.9 eV, which are related to Ti 2p_3/2_ and Ti 2p_1/2_ of TiO_2_. Similarly, there are two peaks that can be confirmed at 458.4 and 464.1 eV in the high‐resolution Ti 2p spectrum of TCNF (Figure S6b, Supporting Information).^[^ ^44^ ^]^ In the high‐resolution Ni 2p spectra of NTCNF (Figure 1i), two characteristic peaks at 852.89 and 871.70 eV correspond to the Ni 2p_1/2_ and Ni 2p_3/2_ of Ni element of NTCNF, respectively.^[^ ^20^ ^]^ Two evident peaks with respect to Ni^2+^ and shakeup satellites are located at 857.60 and 877.50 eV, due to the bonding of Ni with the oxide from surface oxidation.^[^ ^45^ ^]^ The above XRD, XPS, and morphology analysis results demonstrate that the bifunctional amorphous TiO_2‐_ *_x_* @Ni‐CNF electrocatalyst with selective pair sites was successfully synthesized. Figure 1j shows the O 1s core‐level XPS spectra of the TCNF and NTCNF samples. The peak at 531.5 eV is ascribed to oxygen defect sites with low oxygen coordination, whereas the peak at 529.9 eV belongs to the Metal‐O bond.^[^ ^46^ ^]^ Both TCNF and NTCNF have distinct characteristic peaks at 531.5 eV, which are caused by the absence of some oxygen atoms in amorphous TiO_2‐_ *_x_* during low‐temperature nucleation.

**Figure 2:**
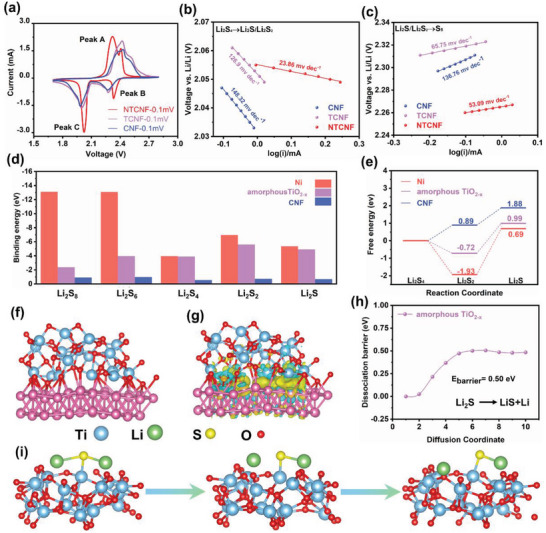
a) CV profiles of S/NTCNF, S/TCNF, and S/CNF electrodes at a scan rate of 0.1 mV s^−1^. b,c) Tafel plots of peak C and peak A of NTCNF. d) Adsorption energies of CNF, TCNF, and NTCNF for Li_2_S*_n_* (*n* = 1, 2, 4, 6, 8). e) Gibbs free energy change of Li_2_S nucleation. f) Schematic illustration of atomic bonding of the amorphous TiO_2‐_ *_x_* @Ni hybrid. g) A side‐top view of the charge density difference of amorphous TiO_2‐_ *_x_* @Ni. Cyan and yellow areas indicate electron depletion and accumulation, respectively. h) Energy barrier of the decomposition of Li_2_S on amorphous TiO_2‐_ *_x_*. i) Decomposition pathway of Li_2_S on the surface of amorphous TiO_2‐_ *_x_*.

For investigating the difference in the electrocatalytic performance of Li–S cells based on different cathodes, coin‐type batteries under a sulfur loading of about 1.5 mg cm^−2^ are assembled. **Figure** **2**a presents their cyclic voltammetry (CV) profiles collected in the voltage window of 1.7–2.8 V at a scan rate of 0.1 mV s^−1^. There are two typical cathodic peaks at 2.27–2.33 V and 1.98–2.02 V ascribed to the conversion of S_8_ to soluble polysulfide intermediates and further reduction to insoluble Li_2_S, respectively. Meanwhile, the anodic peak at 2.32–2.43 V represents the reverse conversion of Li_2_S to S_8_.^[^ ^47^ ^]^ Note that the S/NTCNF electrode exhibits the largest peak current and the lowest reaction polarization in comparison with the other two electrodes, suggesting expedited sulfur redox kinetics originating from bifunctional electrocatalytic pair sites formed by electron‐rich Ni nanoparticles and amorphous TiO_2‐_ *_x_*.^[^ ^26^ ^]^ To further reveal the enhanced electrocatalytic activity of NTCNF host materials on the liquid–liquid conversion of LiPSs, Li_2_S_6_ symmetric cells with NTCNF, TCNF, and CNF electrodes as both working and counter electrodes are assembled (Figure S7, Supporting Information). All the CV curves at the scan rate of 0.1 mV s^−1^ exhibited two pairs of redox peaks. Of note, the NTCNF symmetric cell displays a smaller reaction polarization and higher current response than those of TCNF and CNF symmetric cells,^[^ ^48^ ^]^ confirming an enhanced electrocatalytic ability due to the synergistic effect of Ni nanoparticle and amorphous TiO_2‐_ *_x_* heterogeneous pair sites.

The diffusion behavior of Li‐ion in the S cathode is a crucial step in the kinetics of redox conversion of polysulfides. In order to evaluate Li‐ion diffusion properties, CV profiles under different scan rates from 0.1 to 0.5 mV s^−1^ are delivered in Figure S8, Supporting Information, and then the Li‐ion diffusivity can be estimated by the Randles–Sevcik equation.^[^ ^25^ ^]^ All fitted slopes of the NTCNF electrode are the steepest in peak A, peak B, and peak C, demonstrating the fastest migration/diffusion of Li‐ion and rapid LiPSs redox kinetics (Figure S9, Supporting Information). To get insight into the improvement of amorphous TiO_2‐_ *_x_* @Ni heterogeneous pair sites toward sulfur electrochemistry, electrochemical impedance spectra (EIS) is measured. As shown in Figure S10a, Supporting Information, the Nyquist curves of the fresh battery used in the S/NTCNF cathode display a semicircle with the smallest diameter in the high‐frequency region, which represents the charge transfer resistance (*R*_ct_) at the solid electrolyte interface. After the batteries cycle 20 times at 0.5 C, another semicircle appeared after *R*_ct_ related to the mass transfer resistance of Li_2_S_4_ and ions/electrons during Li_2_S nucleation (Figure S10b, Supporting Information).^[^ ^8^, ^25^ ^]^ The Nyquist curve results before and after cycling demonstrate that NTCNF electrodes can accelerate charge transfer on the electrocatalytic sites to enhance the interfacial redox reaction of sulfur species. To reveal the advantage of this bifunctional electrocatalyst toward polysulfide conversion in‐depth, potentiostatic electrochemical deposition tests were performed by using different catalytic materials (Figure S11, Supporting Information). Compared with TCNF and CNF electrodes, the NTCNF electrode exhibits earlier and larger response currents, as well as higher Li_2_S electrodeposition capacity at a constant potential of 2.05 V. This result indicates that the amorphous TiO_2‐_ *_x_* @Ni heterogeneous pair sites possess excellent electrocatalytic activity toward LiPSs reduction and effectively speeds up the kinetics of Li_2_S nucleation in the sulfur electrochemistry.^[^ ^49^ ^]^

For the sake of accurately revealing the electrocatalytic effect of amorphous TiO_2‐_ *_x_* @Ni heterojunction in working Li–S batteries, Tafel slopes derived from corresponding CV curves (Figure 2a) were fitted based on the reduction process (from Li_2_S_4_ to Li_2_S/Li_2_S_2_) and oxidation reaction (from Li_2_S/Li_2_S_2_ to S_8_).^[^ ^49^ ^]^ As shown in Figure 2b, the fitted Tafel slopes of CNF, TCNF, and NTCNF during the Li_2_S nucleation procedure are 148.32, 126.9, and 23.86 mV dec^−1^, respectively. Notably, when amorphous TiO_2‐_ *_x_* is loaded on the CNF surface, the decrease of Tafel slope value is insignificant (from 148.32 to 126.9 mV dec^−1^), while Ni nanoparticles are loaded, the Tafel slope changes significantly (from 126.9 to 23.86 mV dec^−1^). Intriguingly, the opposite trend occurs during the dissociation of Li_2_S (Figure 2c). After the introduction of amorphous TiO_2‐_ *_x_*, the Tafel slope of the oxidation reaction side has a dramatic decrease (from 136.76 to 65.75 mV dec^−1^), but the introduction of Ni nanoparticles brings little effect (from 65.75 to 53.09 mV dec^−1^). Since smaller Tafel slopes represent a faster reaction kinetics, it strongly indicates that electron‐rich Ni nanoparticle and amorphous TiO_2‐_ *_x_* selectively promote the Li_2_S_4_→Li_2_S and Li_2_S→S_8_ conversion, respectively.^[^ ^17^ ^]^ To shed more light on the effect of amorphous TiO_2‐_ *_x_* and Ni heterogeneous pair sites on enhancing electrocatalytic activity for the sulfur conversion at the atomic level, density functional theory (DFT) calculations were carried out to study nucleation (Li_2_S_4_→Li_2_S) and dissociation (Li_2_S→LiS + Li) reactions of Li_2_S on the surface of pure CNF, amorphous TiO_2‐_ *_x_*, and Ni nanoparticle. First, the optimized structures and calculated binding energies of CNF, amorphous TiO_2‐_ *_x_*, and Ni for Li_2_S_8_, Li_2_S_6_, Li_2_S_4_, Li_2_S_2_, and Li_2_S are shown in Figure 2d and Figures S14–S16, Supporting Information, also the model stability of Ni and amorphous TiO_2‐_ *_x_* are verified in Figure S17 and Table S1, Supporting Information. Obviously, the Ni nanoparticle delivers a stronger adsorption capacity toward LiPSs than that of CNF and amorphous TiO_2‐_ *_x_*, which is consistent with the visualized adsorption testing (Figure 4a). Considering that the nucleation of Li_2_S_4_ to form Li_2_S is the rate‐determining and key liquid–solid phase transition step in the SRR process, the reaction free energy of Li_2_S nucleation from Li_2_S_4_ to Li_2_S_2_ and Li_2_S was calculated. As depicted in Figure 2e, the nucleation‐free energy of the conversion process from Li_2_S_4_ to Li_2_S on the Ni surface is 0.66 eV, which is lower than 0.99 and 1.88 eV of amorphous TiO_2‐_ *_x_* and CNF. Intriguingly, in this decisive liquid–solid phase transition step: Li_2_S_4_→Li_2_S_2_, Ni nanoparticle shows a much lower free energy value (−1.93 eV) and is an entropy reduction reaction, indicating that Li_2_S_4_ tends to undergo the reduction reaction on the surface of Ni nanoparticles. Figure 2f,g shows the optimized structure and the charge density difference of amorphous TiO_2‐_ *_x_* @Ni heterojunction with an isosurface value of 5 × 10^−3^ e bohr^−3^, where the yellow and cyan areas represent the accumulation and depletion of electron cloud density, respectively. It is observed that in the amorphous TiO_2‐_ *_x_* @Ni heterojunction, the electrons are accumulated on the Ni side, which is favorable for the SRR reaction on the Ni surface. The SER process is of equal importance in the bidirectional sulfur redox reaction. In terms of Li_2_S dissociation, it is well established that the initial delithiation (Li_2_S→LiS + Li) plays a key role in the entire reaction. The Li_2_S decomposition pathways on the surface of amorphous TiO_2‐_ *_x_*, CNF, and Ni nanoparticles are modeled (Figure 2I and Figures S18 and S19, Supporting Information). As depicted in Figures S17 and S18, Supporting Information, after the dissociation of Li_2_S on the Ni and CNF surfaces, the Li atom still tends to bond with the S atom to form Li_2_S, which indicates that Li_2_S cannot be effectively decomposed on the Ni and CNF surfaces. In contrast, the delithiation process is successfully catalyzed by the amorphous TiO_2‐_ *_x_* (Figure 2i). Figure 2h shows the dissociation barrier of amorphous TiO_2‐_ *_x_*, a very low value: 0.50 eV. It demonstrates that the amorphous TiO_2‐_ *_x_* can effectively boost the Li_2_S dissociation reaction in the SER process by virtue of its close contact with Li_2_S and the enhanced oxidizability caused by oxygen vacancies. These theoretical analyses once again confirm that engineering CNF with both amorphous TiO_2‐_ *_x_* and Ni nanoparticles will allow optimal bidirectional electrocatalytic reaction kinetics of Li_2_S redox.

**Figure 3:**
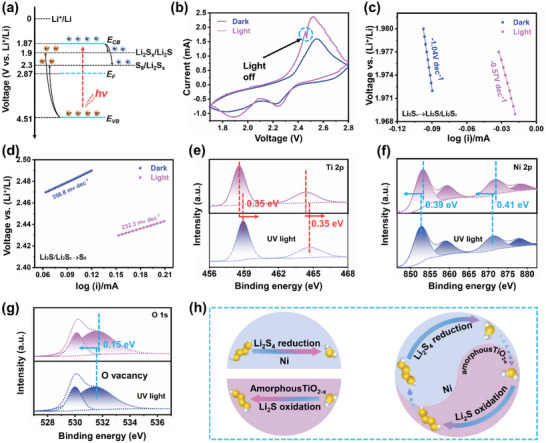
a) Energy diagram of NTCNF and S_8_/Li_2_S versus Li^+^/Li. b) CV curves of the NTCNF Li–S battery at a scan rate of 0.2 mV s^−1^ with and without the illumination (*λ* = 365 nm), the blue circle shows a light switch. c,d) Tafel plots of NTCNF with and without UV light illumination (*λ* = 365 nm). e–g) Ti 2p, Ni 2p, and O 1s ISI‐XPS spectra of NTCNF with and without UV light illumination (*λ* = 365 nm). h) Schematic illustration of one‐directional catalyst (Ni or amorphous TiO_2‐_ *_x_*) and the bidirectional catalyst incorporating Ni and amorphous TiO_2‐_ *_x_*.

In order to locate the electrocatalytic sites and reaction route more accurately in the redox reaction of Li–S battery, ISI‐XPS coupled with the light field is utilized to directly observe the directional transport of photogenerated carriers and the resulting improvement in the redox kinetics of the Li–S battery. In General, the photogenerated electron–hole pairs generated by the electrocatalyst are injected into the coupled light field Li–S battery to amplify the electrocatalytic performance. Whereafter, ISI‐XPS is utilized to observe the transfer routes of photogenerated electrons (strong reducibility) and holes (strong oxidizability) inside the electrocatalyst to further reveal the heterogeneous electrocatalytic pair sites inside the Li–S system. It should be noted that a 365 nm ultraviolet (UV) light with a power of only 5 mW as the light source for avoiding the photothermal effect of infrared light on the electrochemical performance of Li–S batteries. Ultraviolet photoelectron spectroscopy (UPS) with the excitation energy of 21.22 eV as a powerful technique is applied to study the band structure of amorphous TiO_2‐_ *_x_* @Ni‐CNF. As exhibited in Figure S12a, Supporting Information, the cut‐off edge of the high binding energy value (*E*_cutoff_) of NTCNF is 16.41 eV versus the vacuum level (Vac). Hence the work function (*Φ*) value of NTCNF is 4.61 eV. Consequently, the Fermi level (*E*_f_) of NTCNF can be calculated to be −4.61 eV versus the vacuum level (0.17 eV vs NHE). Based on the results above and the Fermi edges in Figure S12b, Supporting Information, the VB position (*E*_VB_) of NTCNF is derived to be 1.47 eV versus NHE (4.51 V vs Li^+^/Li). Figure S12c, Supporting Information, shows the Mott–Schottky (M–S) plot of the amorphous TiO_2‐_ *_x_* @Ni‐CNF heterostructure. The positive slope of the M–S plot indicates the n‐type semiconducting nature of the amorphous TiO_2‐_ *_x_* @Ni‐CNF with the electron as the majority carrier. The flat band potential (*E*_FB_) of the amorphous TiO_2‐_ *_x_* @Ni‐CNF is estimated to be −1.17 V versus Ag/AgCl (≈2.07 V vs Li^+^/Li), which is more positive by ≈0.2 V than that of the CB. The estimated CB and VB potentials of the NTCNF and the relative potentials of the S_8_/Li_2_S are shown in **Figure** **3**a. Clearly, the reaction potentials involved in the entire reaction process of the sulfur in the battery are just located in between the CB and VB of the amorphous TiO_2‐_ *_x_* @Ni‐CNF electrocatalyst. After coupling the light field, the CV profiles of the NTCNF battery with the illumination exhibit higher peak current and lower reaction polarization in the charging and discharging process, suggesting the sulfur redox reaction could be effectively promoted by the amorphous TiO_2‐_ *_x_* @Ni‐CNF bifunctional electrocatalyst with selective pair sites under the light illumination (Figure 3b). Notably, within the blue circle in Figure 3b, the CV current decreases rapidly without light illumination and increases again with illumination. It proves that the enhanced sulfur redox kinetics under illumination comes from the contribution of photogenerated electron–hole pairs in the amorphous TiO_2‐_ *_x_* @Ni heterojunction rather than the photothermal effect. As depicted in Figure S13, Supporting Information, the NTCNF battery with illumination exhibits an exceptionally smaller semicircle diameter than the battery without illumination, confirming the conspicuous enhancement in both ion and electron transport by the illumination. The increase in conductivity should be attributed to the photoconductive effect that the photogenerated electrons concentration increases with the illumination. To precisely analyze the nucleation and dissociation reactions of Li_2_S, Tafel curves of the corresponding redox peaks were plotted (Figure 3c,d). With the light illumination, the Tafel slopes corresponding to the deposition and decomposition of Li_2_S decrease dramatically (−1.04 to −0.57 V dec^−1^ and 356.8 to 232.3 mV dec^−1^). The photogenerated electron–hole pairs originating from amorphous TiO_2‐_ *_x_* @Ni heterogeneous pair sites drive the enhanced redox kinetic performance. Further, the ISI‐XPS have been performed to disclose an authentic pathway of photo‐induced charge transfer in the amorphous TiO_2‐_ *_x_* @Ni heterogeneous pair sites. As shown in Figure 3e, the Ti 2p_3/2_ (458.56 eV) and Ti 2p_1/2_ (464.32 eV) orbitals of NTCNF are shifted to high binding energies by 0.35 eV under UV light irradiation. In contrast, the Ni 2p_3/2_ (852.95 eV) and Ni 2p_1/2_ (871.56 eV) of NTCNF are shifted to the low binding energy by 0.39 and 0.41 eV, respectively (Figure 3f). This binding energy variation indicates that the electron density was decreased on the Ti 2p levels, while it was opposite on the Ni 2p level under UV light irradiation, confirming that the photo‐generated electrons on the CB of amorphous TiO_2‐_ *_x_* in the amorphous TiO_2‐_ *_x_* @Ni heterojunction are transferred to the surface of Ni nanoparticles. Similarly, the decrease in the binding energy of O1s orbital (0.15 eV) with UV light also means that part of the photogenerated electrons is captured by the oxygen vacancies in amorphous TiO_2‐_ *_x_*, which can effectively improve the separation efficiency of photogenerated carriers and simultaneously improve the oxidation ability of amorphous TiO_2‐_ *_x_* (Figure 3g). It means that the photogenerated electrons on the surface of the Ni nanoparticle as the electrocatalytic sites in the reduction reaction sufficiently accelerate the nucleation of Li_2_S during the discharge process. In the subsequent charging session, the photogenerated holes on the amorphous TiO_2‐_ *_x_* oxidation sites accelerate the decomposition process of Li_2_S. The foregoing ISI‐XPS analysis and illumination test results directly verify that the amorphous TiO_2‐_ *_x_* @Ni bifunctional heterogeneous pair sites electrocatalyst loaded on the flexible CNFs can simultaneously promote the reduction of LiPSs and the oxidation of Li_2_S, according to the selective electrocatalytic effects of the amorphous TiO_2‐_ *_x_* @Ni pair sites. The net result is bidirectional LiPSs redox electrocatalysis (Figure 3h). In contrast, a single electrocatalytic site tends to accelerate the sulfur redox reaction in only one direction.

**Figure 4:**
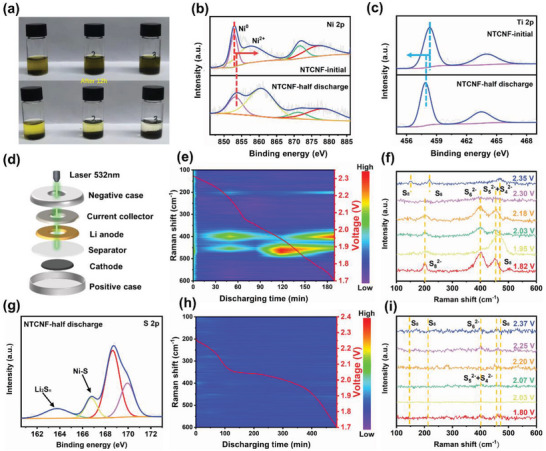
a) Digital photographs of LiPSs adsorption experiment. b,c) Ni 2p, Ti 2p, and S 2p XPS spectra of NTCNF electrode at different discharge states. d) Schematic illustration of a Li–S battery toward in situ Raman test. e,f) In situ time‐resolved Raman spectra obtained during the discharging processes and selected Raman spectroscopy of Li–S cell with S/CNF cathode. g) S 2p XPS spectra of NTCNF electrode at half discharge state. h,i) In situ time‐resolved Raman spectra obtained during the discharging processes and selected Raman spectroscopy of Li–S cell with S/NTCNF cathode, the red curves represent the discharging processes.

In order to more intuitively verify the adsorption effect of the as‐prepared samples for the polysulfides, the visual adsorption experiment of Li_2_S_6_ was carried out in parallel. In an argon‐filled glove box, an equal concentration of 2 mmol mL^−1^ Li_2_S_6_ solution was dropped into three vials containing 30 mg of NTCNF, TCNF, and CNF electrode materials, respectively. As shown in **Figure** **4**a, the color of the solution in the NTCNF samples became clear after standing in the glove box for 12 h. This is attributed to the strong chemical affinity of Ni nanoparticles for LiPSs. In contrast, the color of the liquid in the TCNF sample is slightly yellowish due to the single amorphous TiO_2‐_ *_x_* electrocatalyst being inferior to the synergistic adsorption of amorphous TiO_2‐_ *_x_* @Ni heterojunction. For the CNF‐containing solution, the slight color change for 12 h was because of the infinitesimal interaction with polysulfides. Furthermore, the strong chemical interactions between LiPSs and NTCNF are investigated via XPS tests. XPS spectra were measured at the selected states of the initial and discharge to 2.1 V (half discharge). As depicted in Figure 4b, when the NTCNF cell was discharged to 2.1 V, the peak height of zero‐valent Ni on the Ni 2p orbital decreased with the increase of Ni^2+^ peaks, also a significant upshift by 0.31 eV toward higher BE can be observed. Meanwhile, in the XPS spectrum of the S element (Figure 4g), a new peak at 166.8 eV appeared, which represents the Ni—S bond.^[^ ^20^ ^]^ Compared with NTCNF, only a slight Ti—S bond is detected on the S element spectrum of TCNF (Figure S20, Supporting Information). It fully proves that the electrons transfer from Ni to polysulfides occurs in the reduction reaction of LiPSs, which is related to the charge density difference result shown in Figure 3d. The Ni—S bond formed by the strong interaction between Ni nanoparticles and LiPSs generates a strong chemical anchor effect for polysulfides and accelerates the conversion of polysulfides to Li_2_S. The Ti 2p peak of NTCNF and TCNF shift toward lower BE with regard to that of the initial state (Figure 4c and Figure S21, Supporting Information), testifying the increased electron density located at the Ti metal center and corresponding chemical interaction with LiPSs.^[^ ^25^ ^]^

To explore the shuttling mechanism of LiPSs and the conversion process of sulfur species in‐depth, in situ Raman spectroscopy was executed. The configuration of in operando Li–S cell is exhibited in Figure 4d. The hole is created on the negative case, current collector, and the Li metal anode at the same time to permit laser illumination onto the separator. According to our design, the Raman signal is collected from the separator near the Li‐metal interface, which can accurately provide feedback on the LiPSs deposited on the separator. Figure 4e,f exhibits the time‐resolved Raman spectra during the discharging process of Li–S cells based on the S/CNF cathode at 0.1 C. With respect to the S/NTCNF cathode, three Raman characteristic peaks of S_8_ (located at 150, 217, and 473 cm^−1^) emerge at the beginning of the discharge process (first plateau at ≈2.35 V).^[^ ^25^, ^50^, ^51^ ^]^ With the decrease of the discharge voltage, the peak of S_8_ gradually disappeared, and the peaks at 402 and 454 cm^−1^ belong to S_6_^2−^ and S_5_^2−^ + S_4_^2−^ emerged until the end of the discharge.^[^ ^50^, ^51^ ^]^ During this process, the signal of S_6_^2−^ weakened first, and the signal of S_5_^2−^ + S_4_^2−^ increased, which represented the conversion of the long‐chain polysulfides to short‐chain polysulfides. It indicates that LiPSs are most inclined to deposit on the polypropylene (PP) separator due to severe shuttling behavior. Notably, at the end of the discharge process, the signal of S_6_^2−^ is abnormally enhanced, implying that the severe internal redox reaction of CNF cells is attributed to the severe shuttle effect of soluble polysulfides. For comparison, slight Raman signals of LiPSs are detected on the S/NTCNF cathode throughout the entire discharge process (Figure 4h,i), indicating LiPSs shuttling can be effectively restrained in the discharge process of NTCNF‐based Li–S cell. Additionally, in the discharge curves of CNF in situ Raman cells at high S loadings (>6 mg cm^−2^), the normal discharge plateau of Li–S batteries could no longer be observed. In contrast, NTCNF cells have two distinct discharge platforms for Li–S batteries. These results further demonstrate the role of this selective bifunctional amorphous TiO_2‐_ *_x_* @Ni electrocatalyst in suppressing the polysulfide shuttling and accelerating polysulfide reduction kinetics.

**Figure 5:**
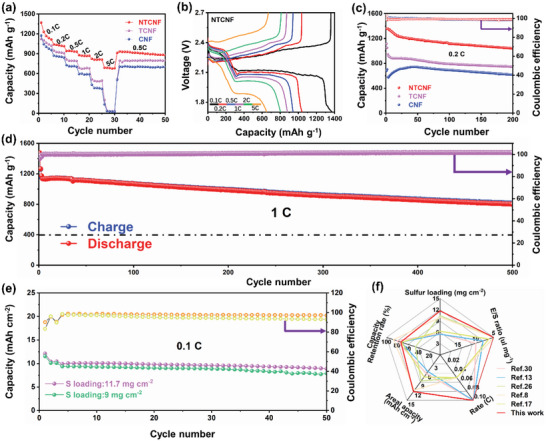
a) Rate performance of S/NTCNF, S/TCNF, and S/CNF cathodes. b) Galvanostatic charge/discharge profiles of S/NTCNF cathode at various current densities from 0.1 to 5 C. c) Cycling performance at 0.2 C for 200 cycles. Cycling performances of NTCNF‐based Li–S cells: d) at a high current density of 1 C and e) at high sulfur area loading. (1 C = 1675 mAh g^−1^). f) Comparison of the performance of NTCNF cathode at the higher sulfur loading with that of various materials reported in the latest works.

**Figure 6:**
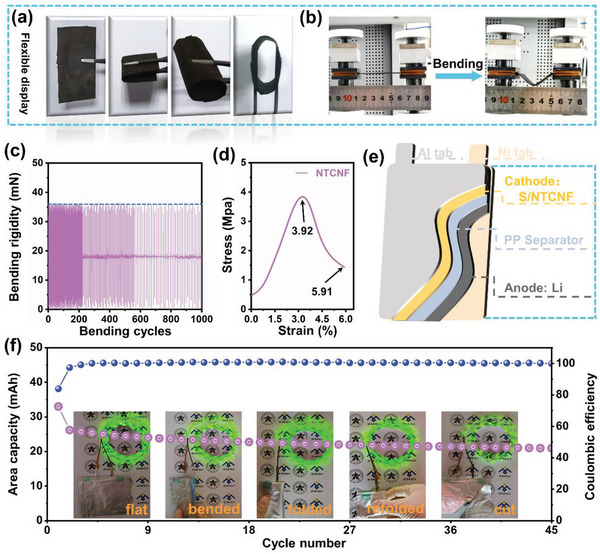
a) The flexible display of NTCNF. b) The profile images of NTCNF before and after bending. c) Bending fatigue test of NTCNF. d) Typical stress–strain curves of NTCNF. e) Schematic illustration of the NTCNF‐based Li–S pouch cell. f) Cycle performance of the pouch cell with NTCNF cathodes at a constant current of 1 mA and demonstration of the pouch cell at various bending states while continuously powering an LED strip.

To reveal the practical effect of the selective bifunctional amorphous TiO_2‐_ *_x_* @Ni electrocatalyst, S/NTCNF, S/TCNF, and S/CNF cathodes with sulfur loadings of about 1.5 mg cm^−2^ were assembled with commercial PP separators and ordinary lithium foil anodes in 2025 coin‐type Li–S cells. In **Figure** **5**a, the S/NTCNF cathode affords extraordinary rate performance, maintaining specific capacities as high as 1371.2, 1041, 942, 879, 824.6, and 689.2 mAh g^−1^ at current densities of 0.1, 0.2, 0.5, 1, 2, and 5 C, respectively. Notably, a high capacity of 934.1 mAh g^−1^ can be recovered when the discharge rate returns from 5 to 0.5 C. The much‐enhanced rate performance by the S/NTCNF cathode demonstrates that the problem of sluggish kinetics of Li_2_S redox was well addressed by the amorphous TiO_2‐_ *_x_* @Ni heterogeneous pair sites, which can selectively catalyze the reduction of polysulfides and the decomposition of Li_2_S. The associated charge/discharge profiles with different current densities are also depicted (Figure 4b and Figures S22 and S23, Supporting Information). The Li–S battery based on the NTCNF electrode preserves two well‐defined voltage plateaus that represent the conversion of S_8_ to LiPSs and the deposition of Li_2_S, although at a high rate of up to 5 C. However, other electrodes, particularly the pristine CNF, suffer from serious capacity degradation and huge polarization with increased current rates. The superb rate capability based on the amorphous TiO_2‐_ *_x_* @Ni heterogeneous pair sites should be ascribed to the high electrical conductivity and superior bifunctional electrocatalytic activity, which can significantly lower the electrochemical resistance and improve the overall redox reaction kinetics of sulfur. Figure S24, Supporting Information, displays the galvanostatic discharge/charge curves of NTCNF, TCNF, and CNF cells at 0.2 C. Both the discharge capacities in the high voltage range from 2.4 to 2.1 V (*Q*_H_) and the capacity in the low discharge voltage range from 2.1 to 1.6 V (*Q*_L_) for NTCNF is much higher than that of TCNF and CNF cells. The higher *Q*_L_/*Q*_H_ proves the better liquid‐to‐solid conversion from the soluble LiPSs to insoluble Li_2_S/Li_2_S_2_ on the S/NTCNF cathode. The voltage hysteresis (Δ*V*, voltage gap between charge and discharge plateaus) is also compared. Apparently, the Δ*V* of the NTCNF cell was much smaller than those two cells, indicating much slighter polarization.^[^ ^52^ ^]^ Reduced polarization resistance conducts better redox kinetics, faster polysulfide conversion, high energy conversion efficiency, and less heat release for safety, particularly at high current density. Moreover, the lower Δ*V* means the smaller total overpotential of the liquid‐to‐solid electrodeposition for S reduction and solid‐to‐liquid conversion for Li_2_S oxidation.^[^ ^53^ ^]^ The higher *Q*_L_/*Q*_H_ and lower Δ*V* originated from the efficient selective electrocatalytic activity of the selective bifunctional amorphous TiO_2‐_ *_x_* @Ni electrocatalyst. The long‐cycle performance of the NTCNF electrode at a low rate of 0.2 C is shown in Figure 5c. The S/NTCNF cathode outputs a high initial specific capacity of 1359.4 mAh g^−1^ at 0.2 C, and still maintains a high specific capacity of 1041.9 mAh g^−1^ after 200 cycles with a Coulombic efficiency above 99%. In contrast, the cycle performance of S/TCNF and S/CNF is much inferior. After 500 cycles at a higher current density of 1 C (Figure 5d), the NTCNF‐enhanced Li–S battery delivered a capacity retention of 803 mAh g^−1^ and a high first‐cycle capacity of 1174.9 mAh g^−1^. The improved cycling performance of NTCNF Li–S batteries are attributed to the polar interaction of Ni nanoparticle with polysulfides and the formation of Ni—S bonds during the discharge process to suppress the shuttling effect, Ni nanoparticles and amorphous TiO_2‐_ *_x_* simultaneously accelerate the LiPSs reduction reaction and the Li_2_S oxidation reaction. In addition, this 3D flexible carbon substrate structure is sufficient to withstand the volume expansion of the sulfur cathode during long‐term cycling, maintaining structural stability. To further demonstrate the promotion effect of NTCNF electrocatalyst on the redox reaction of S species, Li–S batteries with high sulfur loadings of 11.7 and 9 mg cm^−2^ were fabricated (Figure 5e). This Li–S cell with an S loading of 9 mg cm^−2^ can deliver an area‐specific capacity as high as 11.54 mAh cm^−2^ at a density of 0.1 C and lean electrolyte/sulfur (E/S) ratio (1:5), remain 7.91 mAh cm^−2^ after 50 cycles. Even at a higher S loading of 11.7 mg cm^−2^, this NTCNF lithium–sulfur battery still outputs a high areal capacity of 12.1 mAh cm^−2^, which far exceeds most Li–S works. Naturally, with a slow capacity decay rate, the S/NTCNF cathode has a capacity retention of 8.88 mAh cm^−2^ after 50 cycles. Obviously, the acquired areal capacity under high sulfur loading is competitive with the latest publications (Figure 5f). Compared with traditional aluminum‐based current collectors, CNF self‐supporting current collectors have better flexibility, which greatly broadens their application range. As expected, the NTCNF samples still maintain good integrity after folding and crimping, fully demonstrating the high flexibility of NTCNF (**Figure** **6**a). Further, the tensile and flexural properties of NTCNF are shown in Figure 6b–d. Impressively, the 3D nanofiber structure of NTCNF is robust to bear repeated bending tests. After 1000 bending cycles, the flexural strength values of NTCNF are still stable at around 37.5 mN and the bending curve basically remains unchanged (Figure 6c). The tensile strength of NTCNT is 3.92 Mpa with a break elongation of 5.91%, demonstrating the excellent mechanical properties of the NTCNF host (Figure 6d). The outstanding mechanical and electrochemical performance of the NTCNF cathode host inspired us to explore the possibility of further commercial applications. Li–S pouch cell with S loadings of 3 mg cm^−2^ is assembled (Figure 6e). As depicted in Figure 6f, the NTCNF pouch cell exhibits an initial capacity of 24.17 mAh and continued to operate for 45 cycles. Figure S25, Supporting Information, shows the initial charge/discharge curve at 1 mA of Li–S pouch cell with S/NTCNF cathode. The first charge/discharge profile of the pouch cell with S/NTCNF cathode displays minor polarization voltage and exhibits high discharge capacity, manifesting rapid redox kinetics even in the pouch cell. As can be seen from the image of the NTCNF pouch cell lighting up the LED strip in Figure 6f, the NTCNF pouch cells can still work well when bent 90 degrees, folded, and even more extremely folded again, and cut open. It further proves that the 3D flexible structure of the CNFs substrate provides a strong guarantee for the structural stability of the sulfur cathode.

## 3 Conclusion

In summary, we designed a flexible CNF in situ anchored the selective bifunctional amorphous TiO_2‐_ *_x_* @Ni electrocatalyst for Li–S batteries. For the first time, we developed a system that couples the light field and ISI‐XPS to synergistically convert the “black box” battery into a “see‐through” battery for direct observation of the charge transportation, thus revealing that amorphous TiO_2‐_ *_x_* and Ni nanoparticle as the oxidation and reduction sites promote the decomposition and nucleation of Li_2_S, respectively. Also, the theoretical calculation results are consistent with the experimental results, which together provide strong factual support for the selective catalytic mechanism of amorphous TiO_2‐_ *_x_* @Ni heterogeneous pair sites. Such amorphous TiO_2‐_ *_x_* @Ni heterojunction with 3D flexible nanofiber substrate was prepared through an electrospinning method, which combined the merits of excellent catalytic Li_2_S dissociation ability of amorphous TiO_2‐_ *_x_* with the outstanding catalytic LiPSs reduction ability of Ni nanoparticles. The assembled NTCNF‐based Li–S cells deliver good reversibility of 1041.9 mAh g^−1^ for 200 cycles, high‐rate performance (689.2 mAh g^−1^ at 5 C), and durable cycle life for 500 cycles under the current of 1 C with high capacity remaining 803 mA h g^−1^. Moreover, a decent areal capacity of 8.88 mAh cm^−2^ is still maintained at an ultrahigh sulfur mass loading of 11.7 mg cm^−2^ (initial capacity:12.1 mAh cm^−2^) for 50 cycles. Impressively, the pouch cell with S/NTCNF cathode exhibits outstanding flexibility and can still operate continuously under harsh working conditions. Our work demonstrates a feasible strategy for the construction of bifunctional electrocatalysts with selective pair sites that can catalyze the reduction of LiPSs and the decomposition of Li_2_S and provide a universal method to achieve a deeper mechanistic understanding of the bidirectional sulfur electrochemistry.

## 4 Experimental Section

### Synthesis of NTCNF, TCNF, and CNF Electrodes

All chemicals were analytical grade and used without further treatment. First, 0.311 g (CH_3_COOH)_2_·Ni·4H_2_O (nickel acetate tetrahydrate) was dissolved into 15 mL ethanol (C_2_H_5_OH) with continuous stirring and then l.5 mL tetrabutyl titanate (C_16_H_36_O_4_Ti) and 0.15 mL acetic acid (CH_3_COOH) were dropped into the above solution. Finally, 1.5 g PVP was slowly added to the solution. The precursor solution was stirred until the PVP completely dissolved. The 15 kV positive voltage was set for electrospinning. The as‐prepared nanofibers were first stabilized in the air at 250 °C for 2 h. After that, the pre‐oxidized nanofibers were transferred into a vacuum tube furnace and annealed at 600 °C for 60 min in the Ar atmosphere (heating rate was 5 °C min^−1^) to obtain the NTCNF hosts. Similarly, the TCNF and CNF nanofibers were fabricated by removing the Nickel acetate tetrahydrate and the pristine CNF nanofibers were obtained by just adding the PVP.

### Preparation of S/ NTCNF, S/TCNF, and S/CNF Cathodes

The sublimed sulfur, CNTs, and polyvinylidene fluoride (PVDF) were dispersed into *N*‐methyl pyrrolidone (NMP) in a ratio of 8:1:1 and then was sonicated stirring for 12 h to obtain the homogeneous slurry. The S‐contained solution was dripped to 12 mm diameters of the NTCNF (TCNF, CNF) films. After drying at 60 °C for 12 h, the S/NTCNF (S/TCNF, S/CNF) cathodes were successfully prepared. The mass ratio of S: NTCNF (TCNF, CNF) was ≈0.7:1 in conventional coin cells and 1.5:1 at high S loading.

### Visualized Absorption Tests

Li_2_S_6_ solution (0.2 m) was prepared by dissolving a certain amount of Li_2_S and S (the molar ratio is 1:5) in a mixture that consisted of 1,2‐dimethoxyethane (DME) and 1,3‐dioxolane (DOL) (the volume ratio = 1:1). The obtained suspension was vigorously stirred at 60 °C for 12 h and then conversed to Li_2_S_6_ solution. For the visualized absorption tests, 30 mg NTCNF, TCNF, and CNF were added to 5 mL of the as‐prepared Li_2_S_6_ solution, and then observed the color change of the Li_2_S_6_ solution regularly.

### Materials Characterization

The morphologies of the samples were investigated by field‐emission SEM (FESEM, ZEISS SIGMA 500) and TEM (FEI Tecnai G20). Crystal structures of the samples were conducted by XRD using a Rigaku Ultima IV system with Cu *Κα* radiation. The surface chemistry was analyzed by XPS spectra using an AXIS Supra facility while the ISI‐XPS spectra were detected under UV light irradiation at 365 nm. The UPS spectra were recorded with a He I radiation (*hν* = 21.22 eV) source. A platinum wire and Ag/AgCl were employed as the counter electrode and reference electrode, respectively. The electrolyte was a 1 mol L^−1^ Na_2_SO_4_ aqueous solution and the Mott–Schottky test frequency was 1000 Hz.

### Electrochemical Characterization

Prior to electrochemical tests, the S/NTCNF (S/TCNF, S/CNF) electrode (diameter:12 mm) film was directly employed as the cathode of the 2025 Li–S cell with the Celgard 2400 film, 1 m LiTSFI in DOL/DME (1:1 by volume) with 2% LiNO_3_ solution and lithium metal plate was separator, electrolyte, and anode respectively. All Li–S battery assemblies were operated in the glove box filled with high‐purity argon gas. The sulfur mass loading of the cathode electrodes was controlled at about 1.5 mg cm^−2^. The galvanostatic charge/discharge measurement was conducted on a LAND CT2001A system with a voltage window between 1.7 and 2.8 V under various C‐rates (1 C = 1675 mAh g^−1^). The CV and EIS tests were performed on a CHI 660E electrochemical workstation. The EIS measurements began at the open‐circuit potential in the frequency range between 10 mHz and 200 MHz. For a symmetric battery, two identical NTCNF (TCNF, CNF) electrodes were used as the working and counter electrode with 30 µL special Li_2_S_6_ electrolyte. The special Li_2_S_6_ electrolyte consisted of 1 m LiTSFI, 0.1 m LiNO_3_, and 0.2 m Li_2_S_6_ in DOL/DME (1:1 by volume). The CV measurements of the symmetric cell were tested with a voltage window between −1.5 and 1.5 V at a scan rate of 0.1 mV s^−1^. The Li_2_S nucleation experiment was also performed on a CHI 660E electrochemical workstation. For evaluating the liquid–solid reaction kinetics, NTCNF (TCNF and CNF) electrodes, PP, and Li plate were employed as cathode, separator, and anode, respectively.

### DFT Method

DFT calculations were performed by using the Vienna ab initio simulation package (VASP).^[^ ^54^, ^55^ ^]^ The generalized gradient approximation (GGA) with the Perdew–Burke–Emzerhof (PBE)^[^ ^56^ ^]^ functional was utilized to describe the exchange‐correlation energy. Projector‐augmented wave (PAW) methods were employed for the pseudopotentials.^[^ ^57^ ^]^ The energy cutoff for the plane‐wave basis was 400 eV, and the convergence threshold for geometry relaxation was 10^−5^ eV in energy and 0.02 eV Å^−1^ in force. The DFT‐D3 method^[^ ^58^ ^]^ was employed to consider the van der Waals interaction. The *k*‐points in the Brillouin zone were sampled with a 3 × 3 × 1 and 5 × 5 × 1 grid centered for structure optimization and static self‐consistent calculations, respectively. In catalytic reactions, the ease of the reaction was generally assessed by calculating the magnitude of the Gibbs free energy change (Δ*G*) of the chemical reaction, as well as the positive and negative. The Gibbs free energy is calculated as follows

(4) $$G\left(T\right)=E_{\mathrm{DFT}}+E_{\mathrm{ZPE}}+U\left(T\right)-TS$$

where *T* = 298.15 K, *E*_DFT_ is the energy output after VASP calculation, *E*_ZPE_ is the zero‐point energy, *U* is the internal energy of the system, and *S* is the entropy.^[^ ^59^ ^]^

## Conflict of Interest

The authors declare no conflict of interest.

## Supporting information

Supporting Information

## Data Availability

Research data are not shared.
