# Supplementary material for: Revealing the Selective Bifunctional Electrocatalytic Sites via In Situ Irradiated X‐Ray Photoelectron Spectroscopy for Lithium–Sulfur Battery
Source: Adv Sci (Weinh). 2023 Jan 16;10(8):2206786. doi: 10.1002/advs.202206786 (PMC10015878; doi:10.1002/advs.202206786)
Supplement: Supplementary file 1 — Supporting Information [file ADVS-10-2206786-s001.pdf]

Supporting Materials for

Revealing the selective bifunctional electrocatalytic sites via in-situ irradiated X-ray photoelectron spectroscopy for lithium-sulfur battery

Pengpeng Zhang[1,2], Yige Zhao[1], Yukun Li[1], Neng Li[3], S. Ravi P. Silva[1,2,4], Guosheng Shao[1,2], Peng Zhang[1,2*]

1 State Centre for International Cooperation on Designer Low-Carbon & Environmental Materials (CDLCEM), Zhengzhou University, 100 Kexue Avenue, Zhengzhou 450001, China

2 Zhengzhou Materials Genome Institute (ZMGI) Zhengzhou 450001, China

3 State Key Laboratory of Silicate Materials for Architecture, Wuhan University of Technology, Wuhan, China

4 Nanoelectronics Center, Advanced Technology Institute, University of Surrey, Guildford, UK

**Corresponding Authors*

Email: Zhangp@zzu.edu.cn (Peng Zhang)

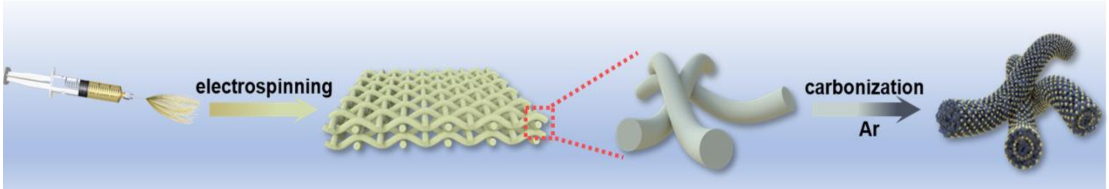

Fig. S1. Schematic illustration of the synthetic procedures for NTCNF electrode.

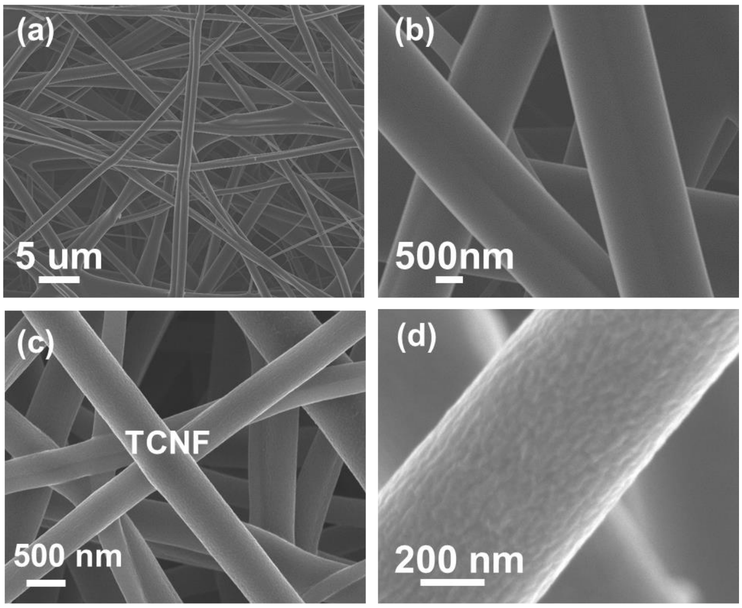

Fig. S2. SEM images: a,b) CNF. c,d) TCNF.

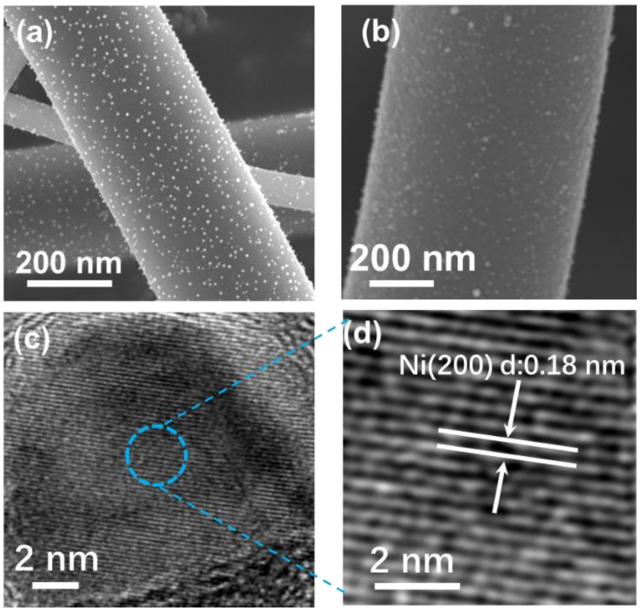

Fig. S3. a, c, d) HRTEM images of NTCNF. b) The SEM image of **Fig. 1g**.

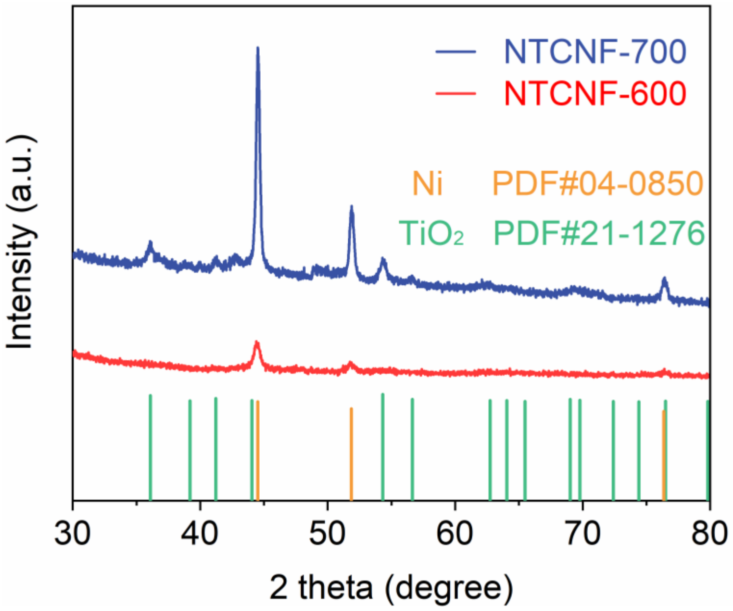

Fig. S4. XRD patterns of NTCNF-600 (NTCNF) and NTCNF-700.

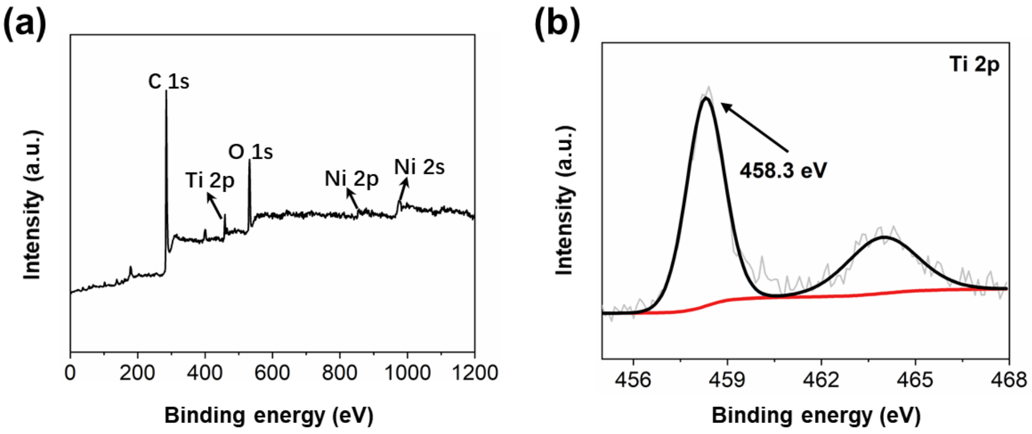

Fig. S5 a) Survey XPS of NTCNF. b) XPS Ti 2p spectrum of NTCNF.

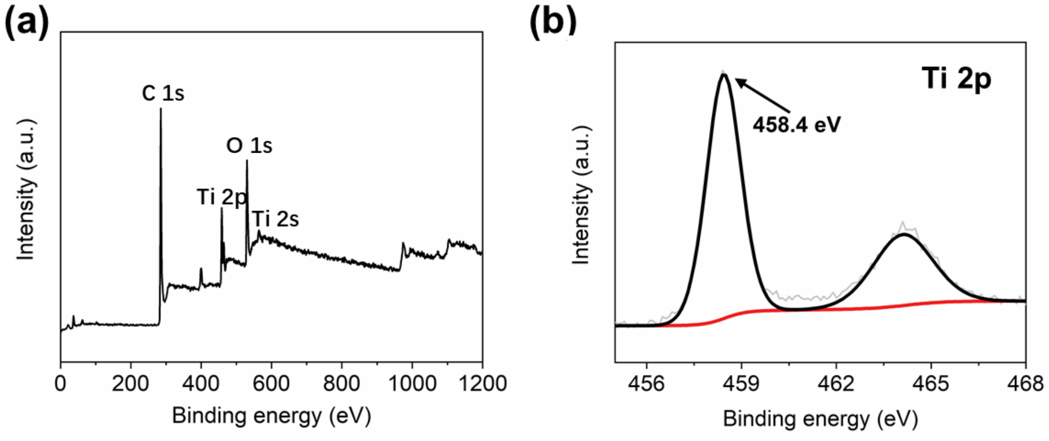

Fig. S6. a) Survey XPS of TCNF. b) XPS Ti 2p spectrum of TCNF.

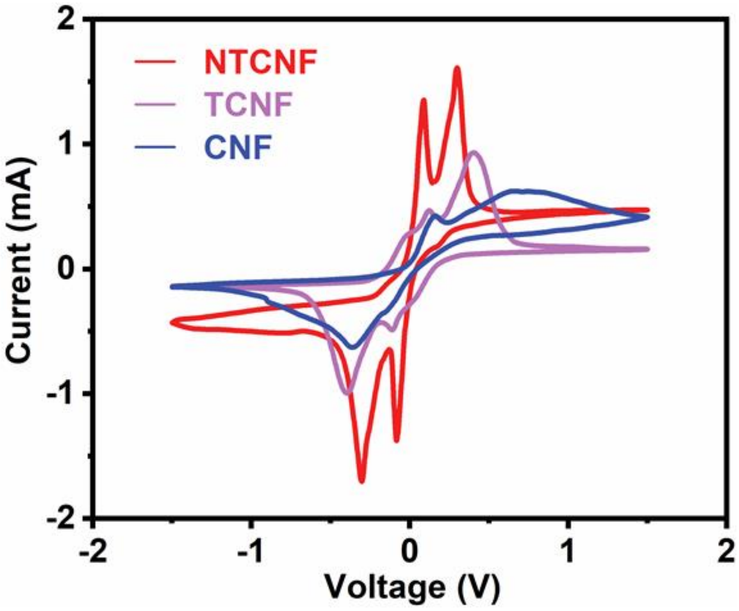

Fig. S7. CV of symmetric batteries with NTCNF, TCNF, and CNF electrodes and Li_2S_6 electrolyte at a scan rate of 0.1 mV s^{-1}.

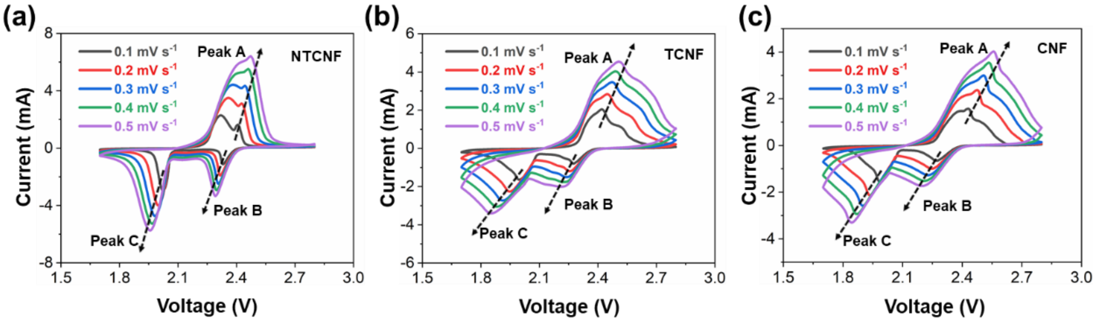

Fig. S8. CV profiles of a) S/NTCNF, b) S/TCNF and c) S/CNF.

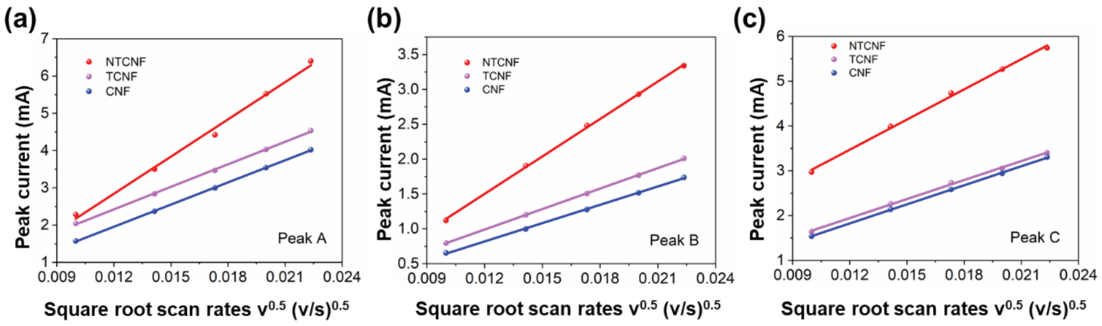

Fig. S9. Plots of CV peak current of a) anodic oxidation process (Peak A: $Li_2S_2/Li_2S \rightarrow S_8$). b) first cathodic reduction process (Peak B: $S_8 \rightarrow Li_2S_n$) and c) second cathodic reduction process (Peak C: $Li_2S_n \rightarrow Li_2S_2/Li_2S$) vs. square root of the scan rates.

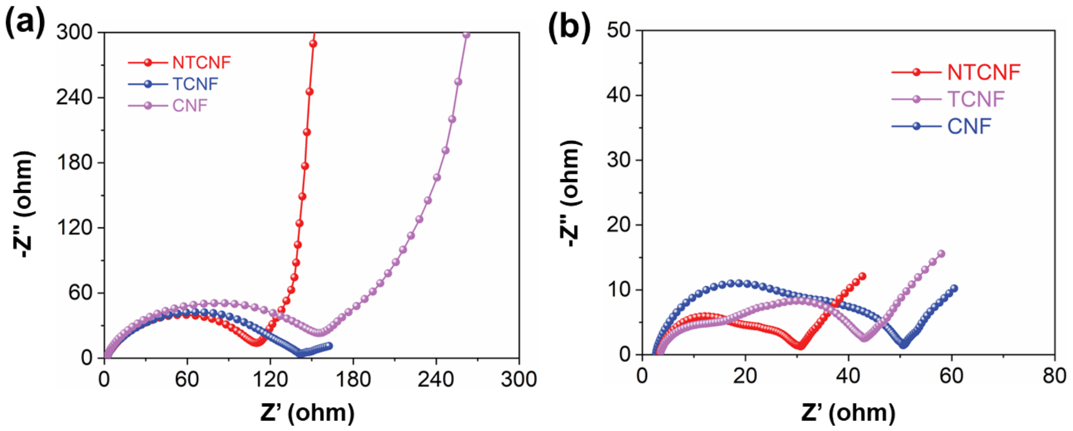

Fig. S10. a, b) Nyquist plots of S/NTCNF, S/TCNF and S/CNF electrodes at first cycle and after 20 cycles at 0.5 C.

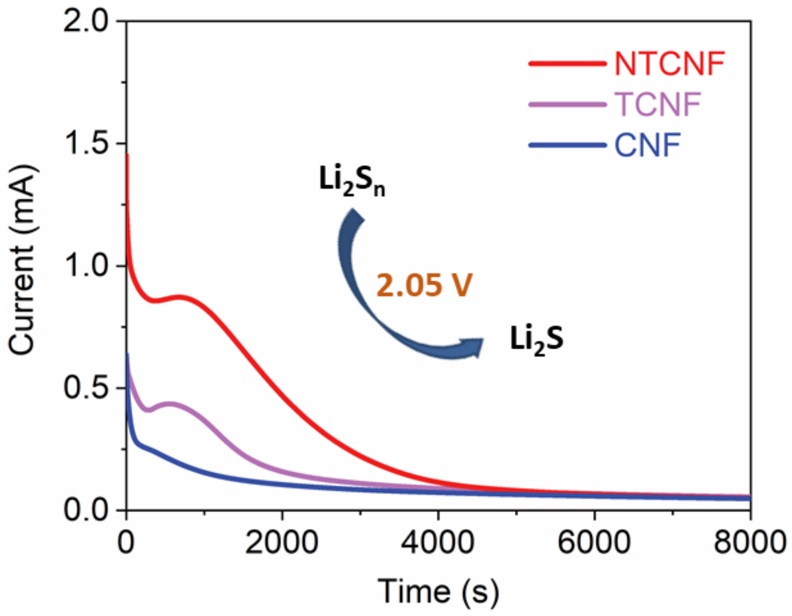

Fig. S11. Chronoamperometry curves of the electrodes with Li_2S_6 electrolyte under a constant over potential of 2.05 V.

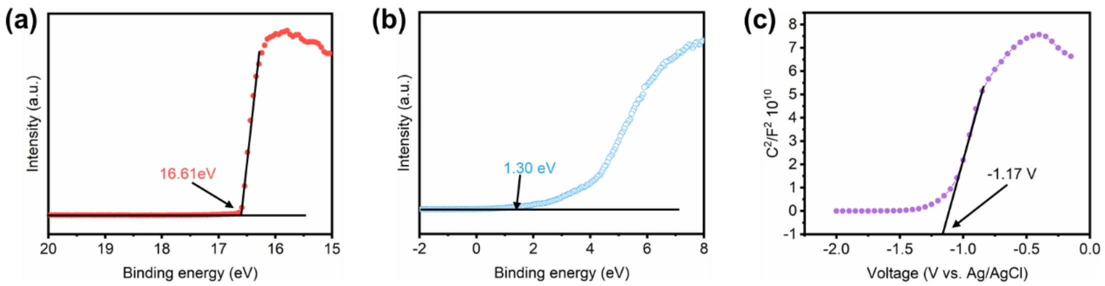

Fig. S12. a, b) UPS spectra of NTCNF. c) Mott-Schottky plot of NTCNF.

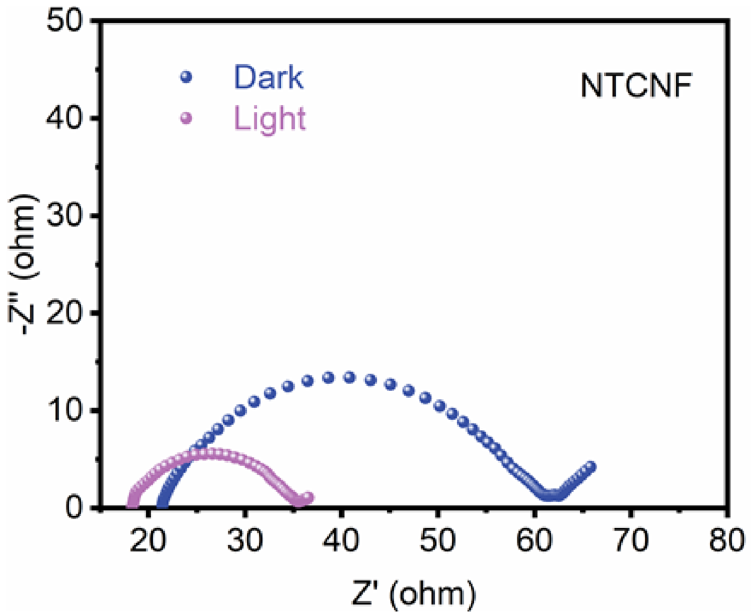

Fig. S13. EIS spectra of NTCNF with and without illumination (λ=365nm).

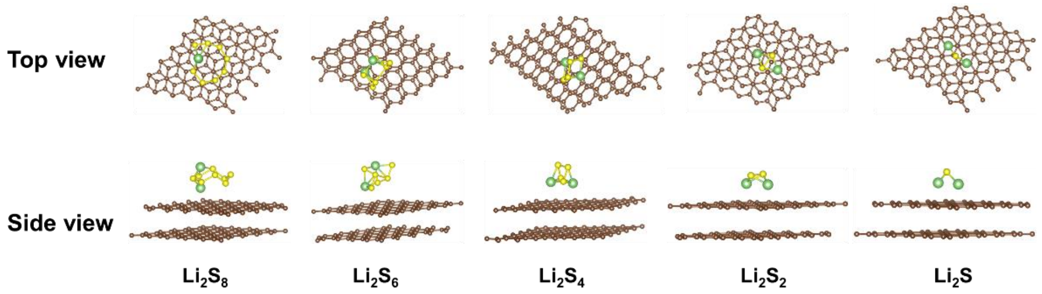

Fig. S14. The optimized adsorption structures of Li_2S_n (n = 1, 2, 4, 6, 8) on CNF surface.

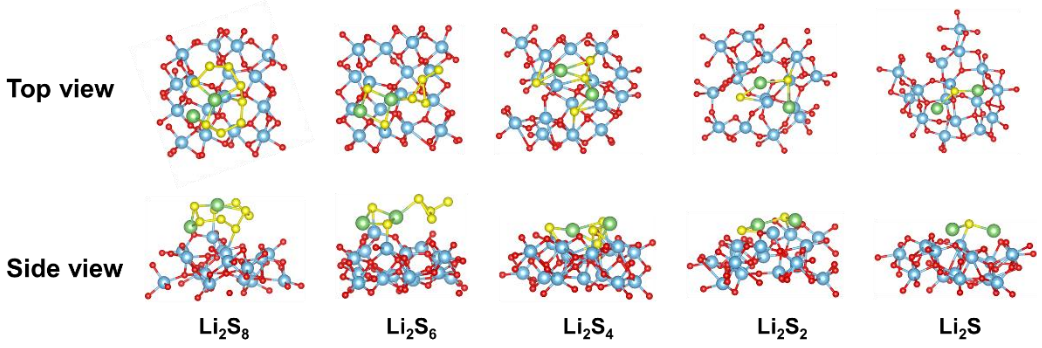

Fig. S15. The optimized adsorption structures of Li_2S_n (n = 1, 2, 4, 6, 8) on amorphous TiO_{2-x} surface.

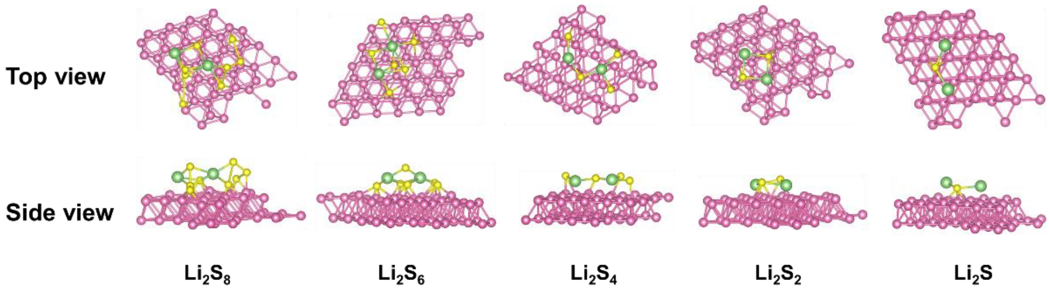

Fig. S16. The optimized adsorption structures of Li_2S_n (n = 1, 2, 4, 6, 8) on Ni surface.

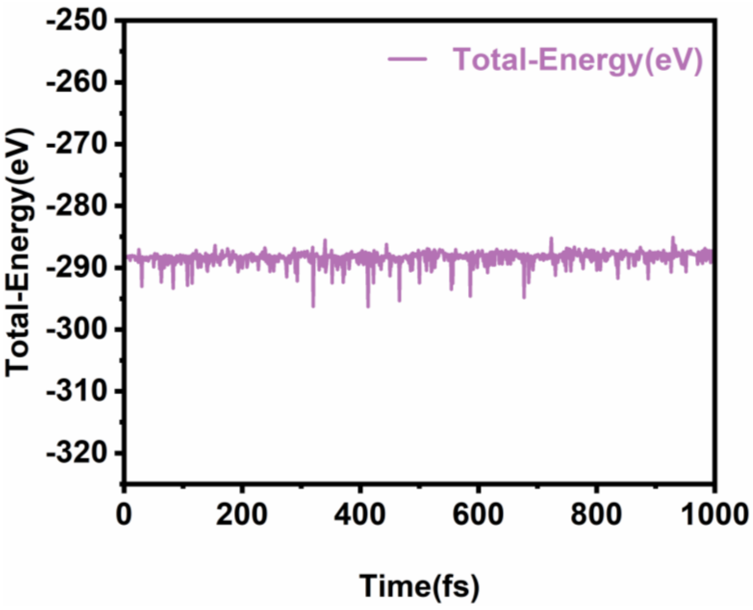

Fig. S17. Energy fluctuation of amorphous TiO_{2-x} with essentially stable molecular dynamics simulation process.

Note: the rutile phase TiO_2 was obtained and its (101) crystal plane was intercepted, and its chemical formula is $Ti_{16}O_{32}$. Then two oxygen atom vacancies are constructed on its surface with the chemical formula $Ti_{16}O_{30}$. For TiO_2 containing O defects, molecular dynamics simulations from 0 K to 1000 K were performed and used to obtain amorphous TiO_{2-x}. After a period of molecular dynamics simulation, the energy of TiO_2 fluctuates within a certain range and the structure remains basically unchanged, and a basically stable amorphous TiO_{2-x} is obtained. Finally, the obtained basically stable amorphous TiO_{2-x} is fully optimized in structure to release the internal stress and obtain stable amorphous TiO_{2-x}.

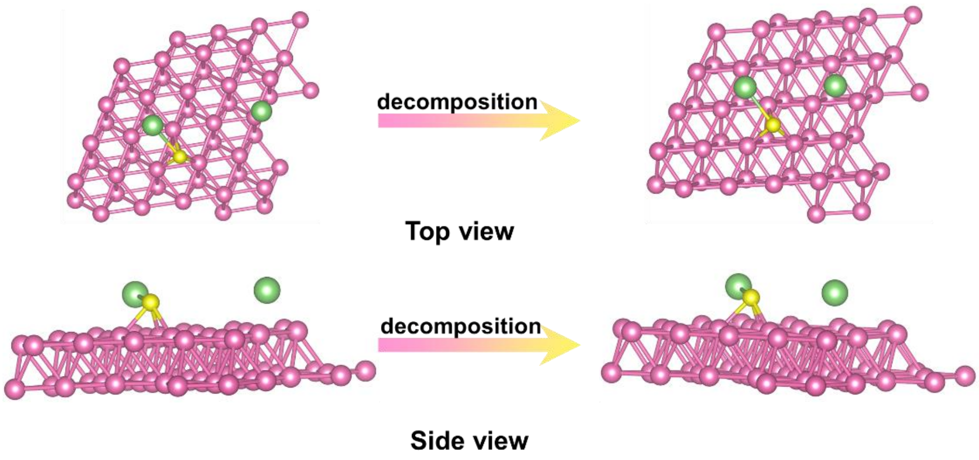

Fig. S18. The decomposition path way of Li_2S on the surface of Ni nanoparticle. Note: Li atom still tends to bond with S to form Li_2S after the decomposition of Li_2S on the Ni surface.

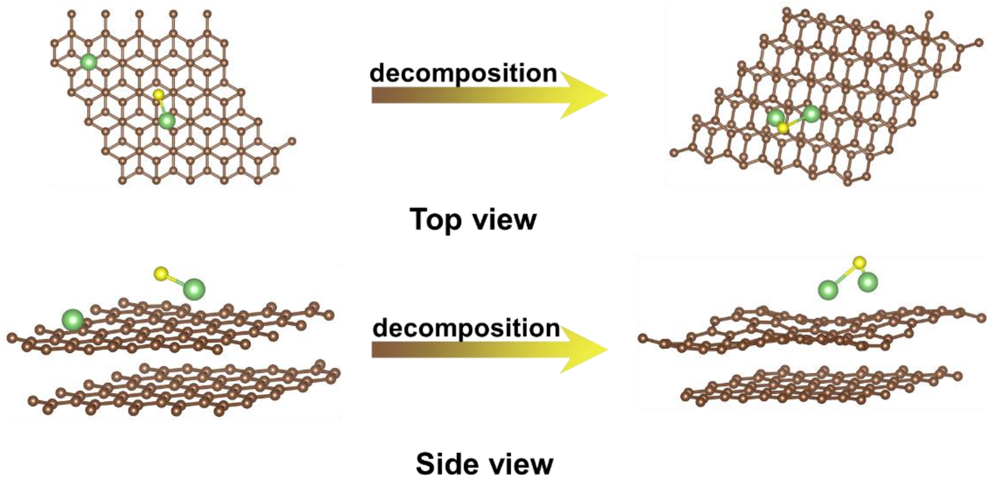

Fig. S19. The decomposition path way of Li_2S on the surface of carbon nanofibers (CNF). Note: Li atom still tends to bond with S to form Li_2S after the decomposition of Li_2S on the CNF surface.

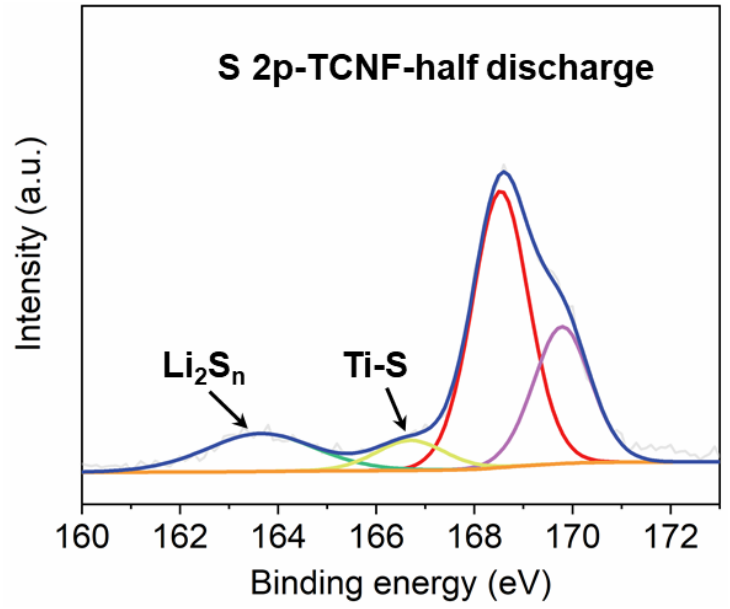

Fig. S20. S 2p XPS spectra of TCNF electrode at half discharge states.

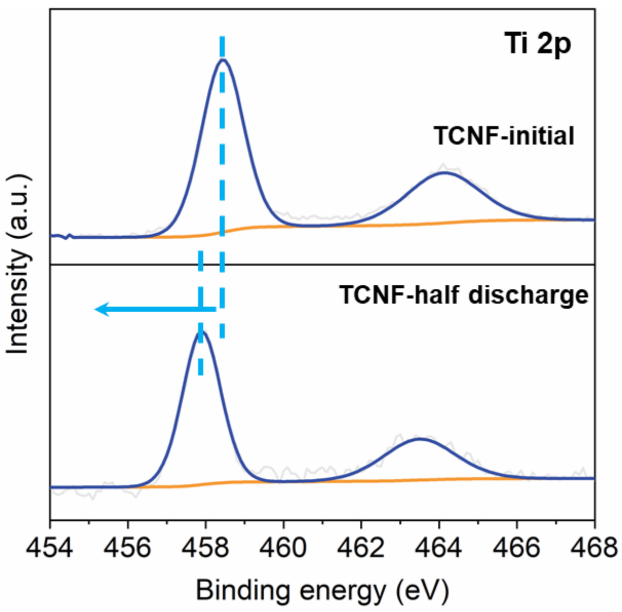

Fig. S21. Ti 2p XPS spectra of TCNF electrode at different discharge states.

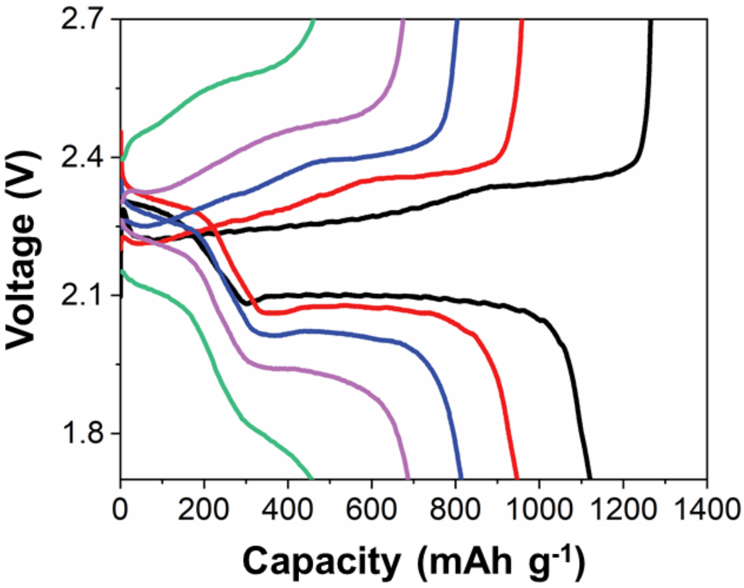

Fig. S22. Galvanostatic charge/discharge profiles of S/TCNF cathode at various current densities from 0.1 C to 2 C.

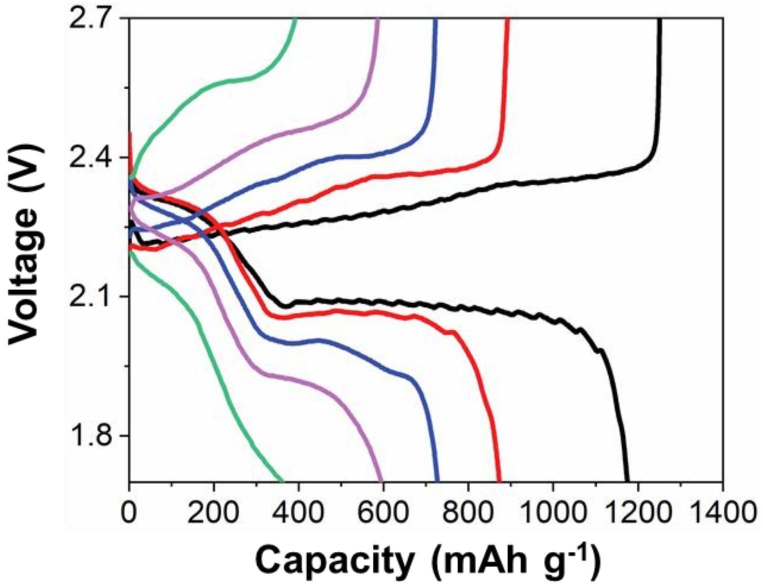

Fig. S23. Galvanostatic charge/discharge profiles of S/CNF cathode at various current densities from 0.1 C to 2 C.

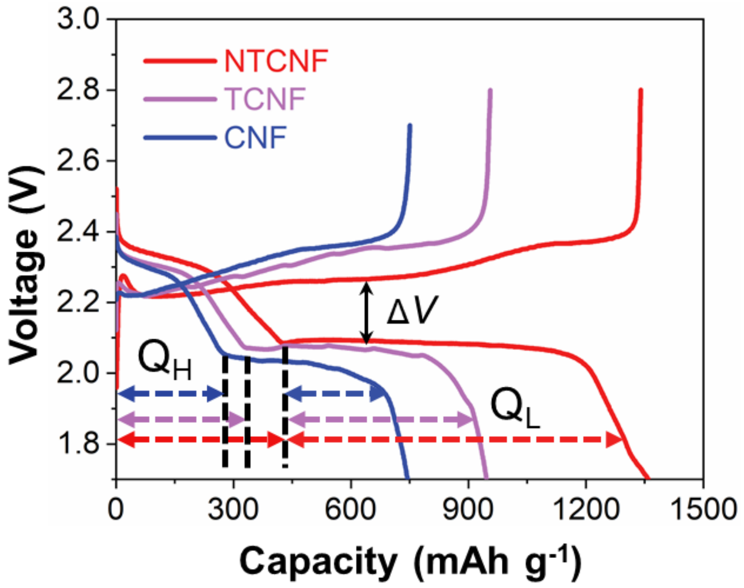

Fig. S24. Galvanostatic discharge-charge profiles at 0.2 C.

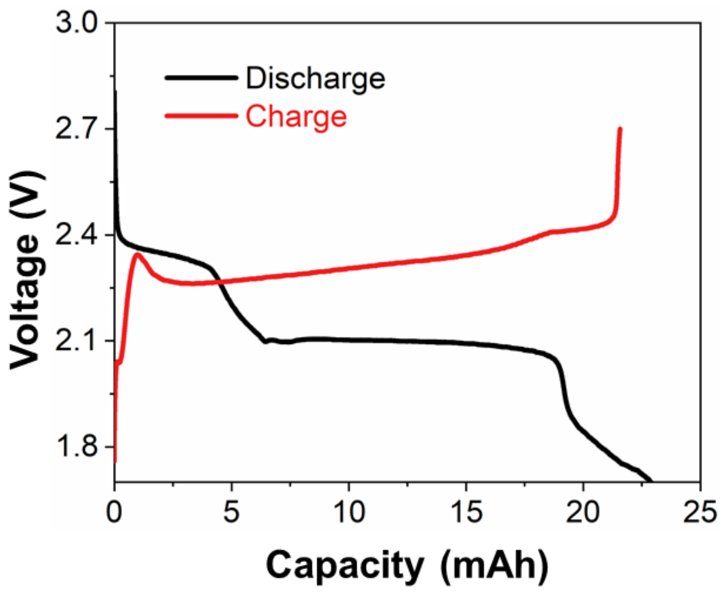

Fig. S25. First cycle galvanostatic charge/discharge profiles of NTCNF based pouch cell.

Table S1: Comparison of the adsorption of polysulfides by nickel Slab models with different thicknesses

**Type**	**Li_2S**	**Li_2S_2**	**Li_2S_6**	**Li_2S_8**
Slab(2layer)	-5.3535201	-6.982779	-13.096786	-13.118693
Slab(4layer)	-5.4685201	-7.097759	-13.294686	-13.225733
difference	2.15%	1.65%	1.51%	0.82%

Note: we did a verification in order to show that the bottom pseudo-surface of the Ni model in Fig.2f and Fig S17 has little effect on the top real surface. In the verification, the thickness of the 001 surface was intercepted as four atomic layers and the bottom two atomic layers were fixed. The adsorption energy of several polysulfides on the surface was mainly calculated. As shown in Table S1, the difference of adsorption energy is less than 3%, that is, the bottom pseudo-surface has little effect on the top real surface. The overall structure of the model remains basically unchanged in the structural optimization, which proves that the Ni model is structurally stable.
